# Genome sequence of an avian paramyxovirus serotype 14 strain isolated in a chicken in South Korea

**DOI:** 10.1128/mra.00541-25

**Published:** 2025-08-11

**Authors:** Sungsu Youk, Andrew Y. Cho, Young-Ki Choi

**Affiliations:** 1 Department of Microbiology, College of Medicine, Chungbuk National University 34933, Cheongju, South Korea; 2 Avian Disease Laboratory, College of Veterinary Medicine, Konkuk University 34965, Seoul, South Korea; Katholieke Universiteit Leuven, Leuven, Belgium

**Keywords:** avian paramyxovirus 14, chicken, live poultry market

## Abstract

An avian paramyxovirus serotype 14 virus was isolated from a chicken in a Korean live poultry market. The full genome shared 91.1% and 88.4% identity with Japanese and Chinese strains. The hemagglutinin–neuraminidase sequence lengths varied due to the different positions of the stop codons, while the total genome length was identical.

## ANNOUNCEMENT

Avian paramyxoviruses (APMVs), members of the subfamily Avulavirinae (family Paramyxoviridae), comprise three genera: *Orthoavulavirus*, *Paraavulavirus*, and *Metaavulavirus*. Their non-segmented, negative-sense RNA genome encodes six canonical proteins (N, P, M, F, HN, and L) and two accessory proteins via RNA editing (1, 2). To date, 22 serotypes have been identified (3). Of these serotypes, formerly APMV serotype 14 (APMV-14), *Metaavulavirus japanense* was first reported in a migratory waterfowl in Japan (4). Here, we report the complete genome sequence of an APMV-14 strain in South Korea.

Trachea and cloacal tissue samples were collected on 20 October 2017, from a chicken purchased and slaughtered for meat at a live poultry market in Cheongju city (IACUC approval no. CBNUR-1041-16). The chicken appeared healthy with no clinical signs. A pooled tissue homogenate was inoculated into 10-day-old embryonated chicken eggs, and the resulting allantoic fluid was subjected to a hemagglutination assay following a standard method (5). Viral RNA was isolated using the TRI reagent and Direct-zol RNA MiniPrep Plus kit (Zymo Research, USA). Complement DNA was synthesized using SuperScript IV reverse transcriptase (Invitrogen, Carlsbad, USA) using random hexamers. The cDNA was randomly amplified as previously (6). Libraries were prepared using in-house-designed primer sets and the Illumina platform-based Barcode-Tagged Sequencing kit (Celemics Inc., Seoul, Korea) for whole-genome sequencing on a MiSeq sequencer (150 bp paired-end mode; Illumina, San Diego, CA, USA). All read processing and assembly were performed in Geneious Prime v.2023.0.1 using default settings. Low-quality bases (<Q20) were trimmed, and an initial *de novo* assembly was performed on the 225,404 reads. The resulting consensus sequence was queried against the NCBI database using BLAST, which identified closely related APMV-14 strains. All raw reads were then mapped to the APMV-14 reference genome (KX258200), resulting in 181,949 mapped reads. The mean coverage depth across the complete genome was approximately 1,203×, with 100% of the reference genome covered. The assembly showed that 95.7% of bases had scores of >Q30. Annotation and pairwise comparison were based on publicly available APMV-14 sequences: the APMV14/duck/Japan/11OG0352/2011 (Dk/Japan) (KX258200) and APMV14/chicken/Fujian/2160/2020 (Ck/China) (MZ351191).

The isolate, designated APMV14/chicken/South Korea/L4/2017 (Ck/Korea) had a genome size of 15,444 nucleotides following the “rule of six” (7) and contained six open reading frames, with an overall GC content of 42.4%. Genome comparison showed nucleotide identity of 91.1% and 88.4% to Dk/Japan and Ck/China. The furin cleavage motif of the F protein is a key determinant of pathogenicity in APMV serotype 1, which is known to cause highly contagious and often fatal disease in poultry (8). The furin cleavage motif of the F protein in the Ck/Korea and Ck/China strains had an arginine residue at the C-terminus of the F2 protein (TREGR↓L), while the DK/Japan had a Lysine residue (TREGK↓L). The HN protein of Ck/Korea (*n *= 587) was estimated to be longer than Dk/Japan (*n *= 580) but shorter than Ck/China (*n *= 607) due to the different positions of the stop codons. Continued surveillance and infectivity studies are required to monitor the spread and potential pathogenicity in domestic poultry.

## Data Availability

The complete genome sequence of APMV14/chicken/South Korea/L4/2017 was deposited in the GenBank under the accession no. PV643989. Raw sequence reads are available in the Sequence Read Archive under accession no. SRR33854851.
